# Analysis of glucose metabolism outcomes 4–7 years postpartum in women with gestational diabetes mellitus using continuous glucose monitoring maternal risk factors: a Chinese cohort study

**DOI:** 10.3389/fendo.2025.1596717

**Published:** 2025-09-30

**Authors:** Dan Zhao, Ning Yuan, Xin Zhao, Jianbin Sun, Xiumei Xu, Xiaomei Zhang

**Affiliations:** Department of Endocrinology, Peking University International Hospital, Beijing, China

**Keywords:** gestational diabetes mellitus, postpartum period, continuous glucose monitoring, glycemic variability, risk factors

## Abstract

**Background:**

This study investigates glucose metabolism outcomes and glycemic variability in women with gestational diabetes mellitus (GDM) 4–7 years postpartum. It also identifies maternal risk factors for glucose metabolism abnormalities (GMA) to support early prevention strategies.

**Methods:**

A bidirectional cohort study was conducted with 60 women with GDM and 60 without GDM, recruited from Peking University International Hospital between 2017 and 2019. Participants underwent oral glucose tolerance tests at 4–7 years postpartum and were categorized into GMA and normal glucose tolerance groups. Continuous glucose monitoring assessed glycemic variability, and logistic regression identified early pregnancy risk factors for postpartum GMA.

**Results:**

(1) Women with a history of GDM have a higher incidence of GMA 4–7 years postpartum (p< 0.001). (2) They also showed increased cardiovascular risk factors 4–7 years postpartum, including diastolic blood pressure, body fat ratio, and interleukin-6 (p<0.05). (3) Blood glucose variability is significantly higher in all participants with a history of GDM, even in the normal glucose tolerance group. (4) Independent early pregnancy predictors of postpartum GMA included pre-pregnancy body mass index (BMI), the triglyceride-glucose index, and a history of GDM (AUC = 0.870, 95% CI: 0.808–0.931).

**Conclusions:**

Women with a history of GDM are at a higher risk of GMA and glycemic variability 4–7 years postpartum. Pre-pregnancy BMI, the triglyceride-glucose index, and GDM history are strong predictors of postpartum GMA, highlighting the need for early intervention.

Clinical trial registration: China Clinical Trials Registry, identifier ChiCTR2300067592.

## 1 Introduction

Gestational diabetes mellitus (GDM) refers to hyperglycemia first detected during pregnancy that does not meet the diagnostic threshold for diabetes (1, 2). In recent years, the incidence of GDM has been steadily increasing, significantly impacting the long-term metabolic health of both mothers and their offspring. It has become a major global public health concern (3, 4). The Hyperglycemia and Adverse Pregnancy Outcome (HAPO) follow-up study reported that 52.2% of women with untreated GDM developed postpartum glucose metabolism abnormalities (GMA) (5). Studies have shown that women with a history of GDM have a 7-10-fold higher risk of developing postpartum GMA compared to those with normal blood glucose levels during pregnancy (6).

Evidence suggests that chronic low-grade inflammation persists after GDM, during which multiple physiological pathways are activated, exacerbating insulin resistance (IR). This further contributes to endothelial dysfunction, thereby progressively increasing the risk of GMA, hypertension, dyslipidemia, and atherosclerosis (7). This process may persist for several years or even decades, with insidious symptoms that make early detection challenging. In recent years, continuous glucose monitoring (CGM) has been recognized as a sensitive tool for the early detection of glucose metabolism dysregulation, potentially identifying metabolic changes before overt hyperglycemia becomes apparent (8). However, studies combining CGM with the traditional oral glucose tolerance test (OGTT) to assess the long-term prognosis of women with a history of GDM remain limited.

This study aims to evaluate the impact of GDM on postpartum 4–7 years glucose and lipid metabolism, glycemic variability (GV), and cardiovascular risk. Additionally, it seeks to identify maternal risk factors for postpartum GMA in women with GDM, develop a risk assessment model, and formulate early prevention and intervention strategies to provide a scientific basis for postpartum management of women with GDM.

## 2 Materials and methods

### 2.1 Participants

**Figure 1:**
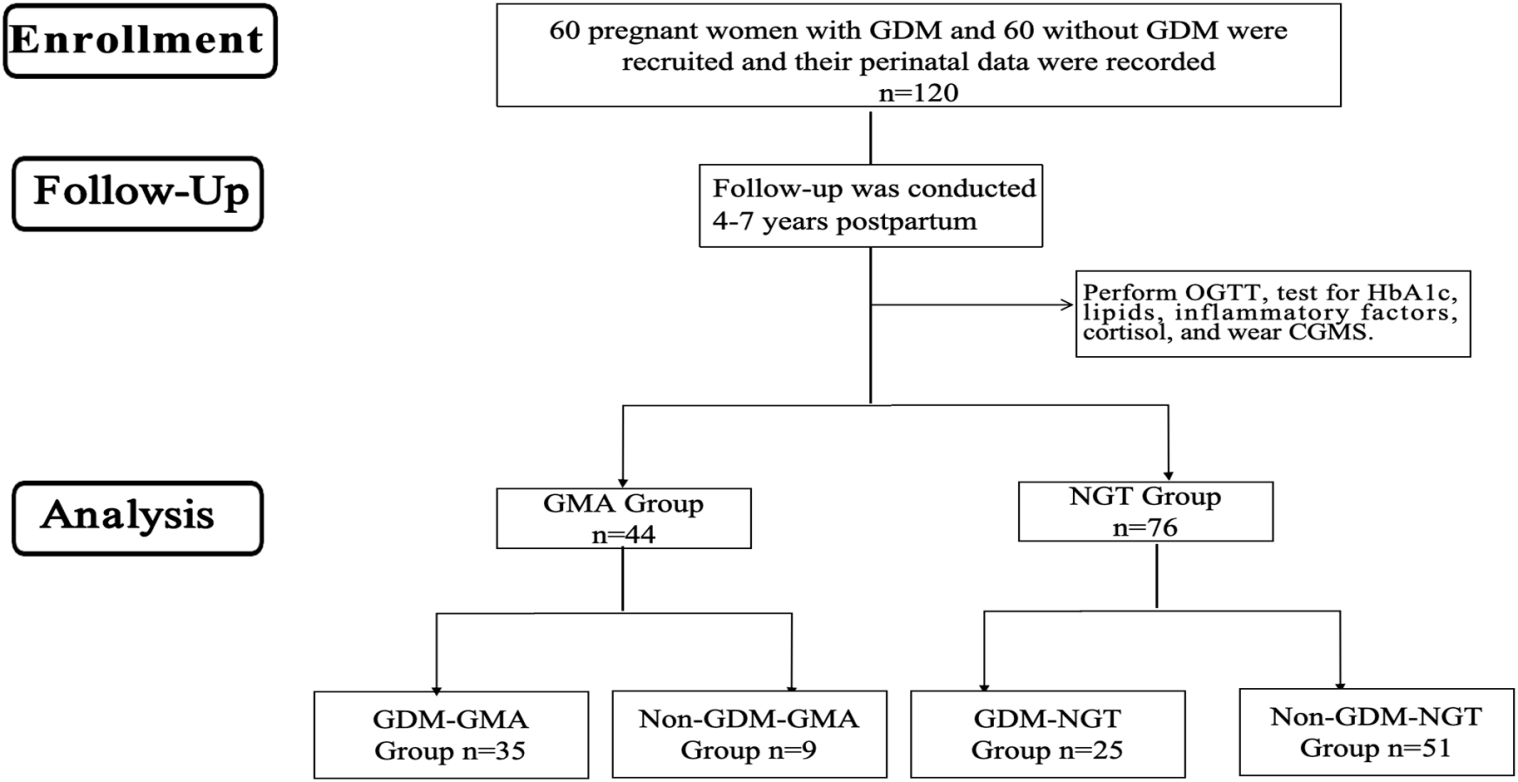
Flow diagram. GDM, Gestational diabetes mellitus; OGTT, oral glucose tolerance tests; HbA1c, glycosylated hemoglobin; CGMS, Continuous glucose monitoring systems; GMA, Glucose Metabolism Anomaly; NGT, Normal Glycemic Tolerance.

This study is a retrospective and prospective two-way cohort study. Based on a prospective cohort of pregnant women established at Peking University International Hospital from 2017-2019, from which GDM and non-GDM women meeting the inclusion and exclusion criteria were screened and matched 1:1 by age, gestational week, and parity, 120 consecutive participants were included to complete 4–7 years of postpartum follow-up (**Figure 1**). Participants underwent OGTT and were categorized into four groups: GDM-GMA group, Non-GDM-GMA group, GDM-normal glucose tolerance (NGT) group, and Non-GDM-NGT group. Additional tests assessed Hemoglobin (HbA1c), blood lipids, inflammatory factors, and cortisol levels, with CGM provided. Inclusion criteria: (1) age ≥18 years; (2) complete perinatal case data; (3) willingness to participate and consent to blood sample collection. Exclusion criteria: (1) pre-pregnancy diabetes and overt diabetes in pregnancy; (2) multiple pregnancies; (3) autoimmune diseases; (4) severe liver/kidney dysfunction; (5) long-term antidepressant/corticosteroid use; (6) use of hypoglycemic medications/insulin during follow-up. The research followed the guidelines of the Strengthening the Reporting of Observational Studies in Epidemiology (STROBE) statement.

### 2.2 Sample size determination

The sample size calculation was based on previous literature parameters: the mean fasting blood glucose levels were 6.2 ± 1.9 mmol/L in the GDM group and 5.0 ± 1.6 mmol/L in the non-GDM group. Setting α = 0.05 (two-sided) and β = 0.10, the required sample size for each group was calculated using PASS 11 software (independent samples t-test) to be 46 cases. Considering the 10% loss-to-follow-up rate, a minimum of 52 cases per group was required after correction. To ensure statistical efficacy, 60 cases per group were finally included in this study.

### 2.3 Perinatal information

Patient perinatal data was based on our previously established cohort (9), which was collected at the time of cohort creation and was available in the electronic medical record system.

### 2.4 Postpartum follow-up information

Basic information was collected from all participants, who were followed up 4–7 years postpartum. Anthropometric measurements, including systolic blood pressure (SBP), diastolic blood pressure (DBP), height, weight, body fat percentage (BFR), waist circumference, and hip circumference, were taken by the same researchers. Blood pressure was measured using an Omron electronic sphygmomanometer (model HEM-7201). Height and weight were measured using a Seca electronic height and weight scale (model 704). BFR was measured using bioelectrical impedance measurement (InBody 750). We gave each participant a retrospective ambulatory glucose monitoring system (Ipro2; Medtronic, Minneapolis, MN, USA) for data collection. Participants underwent fingertip glucose correction twice daily at fasting and bedtime, and the sensor was worn by 15:00 on the day of enrolment and for 7 consecutive 24-hour periods.

Venous serum samples were collected after fasting for 8 hours. The following measurements were made: total cholesterol (TC), triglyceride (TG), high-density lipoprotein cholesterol (HDL-C), low-density lipoprotein cholesterol (LDL-C), Lipoprotein (a)[Lp(a)], and sensitivity C-reactive protein (Hs-CRP). Cortisol (Cor) was collected at 9 am. HbA1c is determined by high-performance liquid chromatography. An OGTT was also performed: 75 g of glucose powder was dissolved in 250 mL of water and administered orally rapidly over 5 minutes. Fasting blood glucose (FBG), fasting insulin (FINS), 2-hour blood glucose, and 2-hour insulin levels after glucose administration were then tested. The above tests were performed in the laboratory of the Department of Laboratory Medicine of Peking University International Hospital, which strictly adheres to the health industry standards of the People’s Republic of China for in-house quality control and has been certified by the National Center for Clinical Laboratories of China for external quality assessment.

Inflammatory factor detection: ELISA was used to detect the inflammatory factors in the serum of the study subjects, including interleukin-6 (IL-6), tumor necrosis factor-α (TNF-α), and tumor necrosis factor-β (TNF-β). The instrument used in this study was the MK3 ELISA kit (Thermo, America), which is Thermo’s high-sensitivity human serum factor kit.

### 2.5 Definitions and calculations

The diagnostic criteria for GDM in this study were based on the criteria of the International Association of Diabetes and Pregnancy Study Groups (10). The history of GDM in the following text refers specifically to GDM diagnosed by OGTT performed at 24–28 weeks of this pregnancy.

Overt diabetes in pregnancy was defined as fasting blood glucose ≥126 mg/dl or 2-hour postprandial blood glucose ≥200 mg/dl (11).

The GMA encompasses Impaired Fasting Glucose (IFG), Impaired Glucose Tolerance (IGT), and Type 2 Diabetes Mellitus (T2DM). The diagnostic criteria for IFG, IGT, and T2DM adhere to the Chinese Guideline for Diabetes Prevention and Treatment and align with the 1999 World Health Organization (WHO) diagnostic standards (12).

Body mass index (BMI) was calculated as: weight (kg)/height^2^ (m^2^). Waist-to-hip ratio (WHR) was calculated as: waist/hip circumference.

TyG Index was calculated $\;\ln\left[\frac{TG\left(mg/dl\right)\times FBG}{2}\right]\;$ ; Homeostasis Model Assessment for IR (HOMA-IR) was calculated as: $\frac{FBG\times FINS}{405}$ ; Homeostasis Model Assessment for β-cell function (HOMA-β) was calculated as: $\frac{20\times FINS}{FBG-3.5}$ ; Matsuda index was calculated as: $\frac{10,000}{{\left[\left(FBG\times FINS\right)\times \left(mean\;glucose\right)\times \left(mean\;insulin\right)\right]}^{1/2}}$ . In the above formula, blood glucose units are mg/dL, and insulin units are μU/mL.

### 2.6 Statistical analysis

Data were analyzed using SPSS 29.0 software. The Kolmogorov–Smirnov test assessed normality. Normally distributed data were expressed using the *mean ± standard (* $\bar{x}$ *± s)*, non-normally distributed data were expressed using the *median (interquartile range)*, and categorical variables were expressed using *absolute numbers and percentages*. Differences between the two groups were compared using the t-test, Mann-Whitney U test, and χ2 test. For multiple groups, one-way ANOVA, covariance ANCOVA, Kruskal-Wallis test, and χ2 test were used, with *post-hoc* comparisons via the Bonferroni method. A p-value<0.05 was considered significant. Binary logistic regression identified early pregnancy risk factors for postpartum glucose metabolism outcomes, and diagnostic performance was evaluated using the receiver operating characteristic (ROC) curve.

## 3 Results

### 3.1 Glucose metabolism outcomes in pregnant women 4–7 years postpartum and baseline

**Figure 2:**
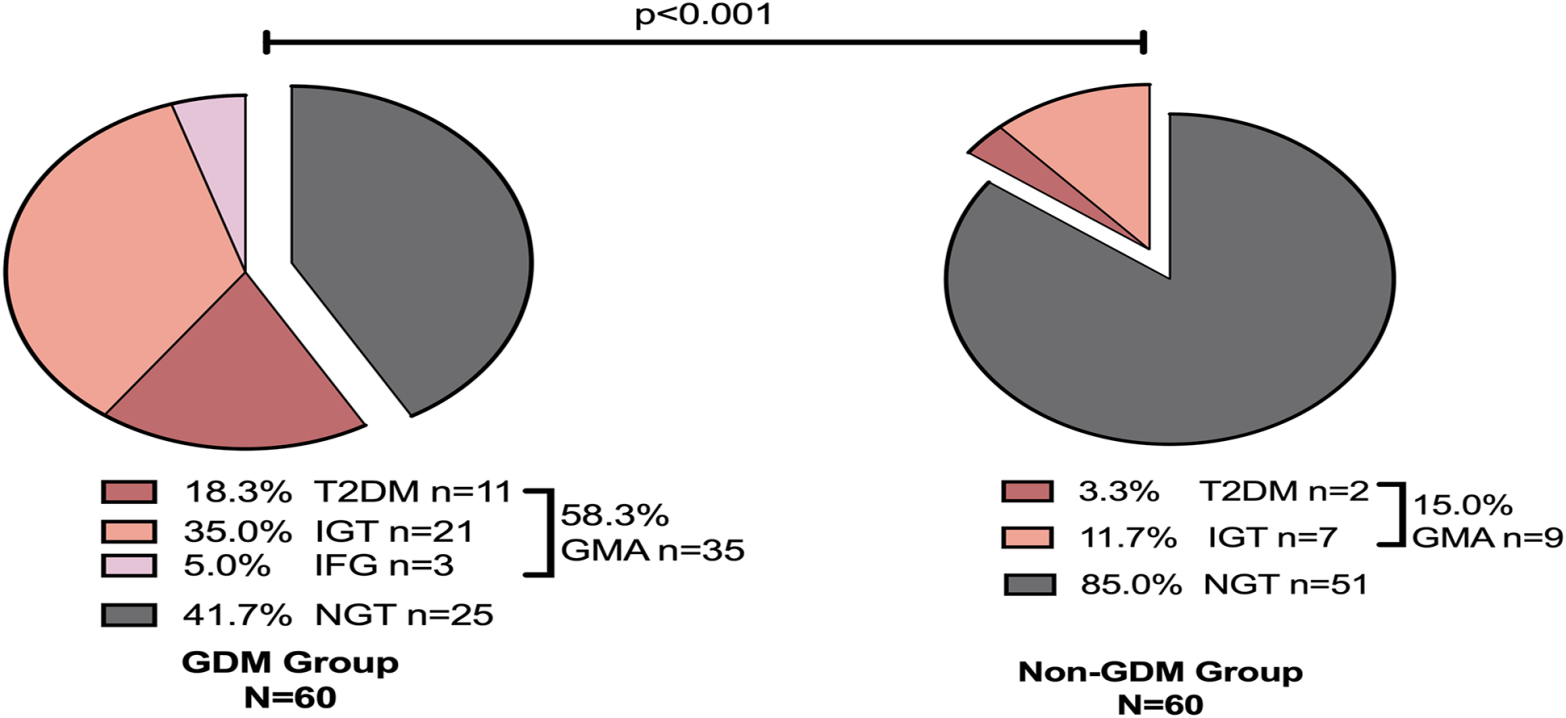
Glucose metabolism outcomes 4–7 years postpartum. GDM, Gestational diabetes mellitus; NGT, Normal Glycemic Tolerance; GMA, Glucose Metabolism Anomaly; T2DM, Type 2 Diabetes Mellitus; IGT, Impaired Glucose Tolerance; IFG, Impaired Fasting Glucose.

A follow-up study was conducted on women with GDM for 4–7 years postpartum, revealing that 58.3% (n=35) developed GMA. Among them, 18.3% (n=11) progressed to T2DM, 35.0% (n=21) developed IGT, and 5.0% (n=3) exhibited IFG, while 41.7% (n=25) maintained NGT. Follow-up in the non-GDM group showed that 15% (n=9) developed GMA. Among them, 3.3% (n=2) progressed to T2DM, 11.7% (n=7) developed IGT, and 85.0% (n=51) maintained NGT. No cases of IFG were reported in this group. Comparatively, the risk of developing GMA in the 4–7 years postpartum period was significantly higher in the GDM group (p<0.001) (**Figure 2**).

**Table 1** summarizes the baseline characteristics of the four study groups (GDM-GMA, Non-GDM-GMA, GDM-NGT, and Non-GDM-NGT). Women with prior GDM, regardless of subsequent glucose metabolism status (GMA or NGT), exhibited significantly higher DBP compared to non-GDM groups[(75.25 ± 12.26,69.88 ± 7.25)vs. (66.55 ± 10.13,66.50 ± 6.99), p = 0.001]. BFR was elevated in the GDM group at 4–7 years postpartum[(34.12 ± 6.21,32.41 ± 5.37)vs. (30.15 ± 7.79,30.03 ± 6.01), p=0.049].IL-6 levels were significantly higher in the GDM group [(3.84 ± 2.27,3.67 ± 1.72)vs. (2.58 ± 1.58,2.52 ± 1.83),p=0.013]. The GDM-GMA subgroup showed elevated TC[(5.16 ± 0.86)vs. (4.79 ± 0.76,4.61 ± 0.62,4.48 ± 0.84),p=0.014], LDL-C[(3.12 ± 0.82)vs.(2.87 ± 0.47,2.73 ± 0.54,2.70 ± 0.72),p=0.040], and Lp(a)[(171.62 ± 99.81)vs.(124.45 ± 79.16,112.86 ± 72.43,111.55 ± 69.76),p=0.018]levels compared to other groups. Subjects with GMA (regardless of GDM history) demonstrated higher FBG compared to the NGT groups [(6.39 ± 2.21, 6.18 ± 2.60) vs. (5.26 ± 0.45,5.01 ± 0.47),p< 0.001]. HbA1c levels differed significantly only between GDM-GMA and Non-GDM-NGT groups (5.95 ± 1.12vs.5.42 ± 0.28,p = 0.001). GMA groups exhibited elevated cortisol levels [(10.50 ± 2.95,9.71 ± 3.42)vs.(8.21 ± 2.59,7.85 ± 3.80),p< 0.05] and increased IR indices:HOMA-IR [(4.84 ± 2.85,3.50 ± 2.76)vs.(2.38 ± 1.66,2.51 ± 1.50), p=0.004], TyG index [(2.08 ± 1.10,1.87 ± 1.22)vs.(1.41 ± 0.93,1.43 ± 0.91),p=0.019], and Matsuda index [(6.95 ± 4.35,9.59 ± 5.47)vs.(13.75 ± 7.46,15.01 ± 7.46),p<0.001].

**Table 1:** Baseline characteristics in pregnant women 4–7 years postpartum.

Characteristic	GDM-GMA	Non-GDM-GMA	GDM-NGT	Non-GDM-NGT	*F*	*P*
Age-offspring(years)	5.57 ± 0.96	5.67 ± 1.00	6.00 ± 1.04	5.98 ± 0.98	1.359	0.208
SBP (mmHg)	116.08 ± 17.61	109.11 ± 9.07	110.36 ± 10.26	109.02 ± 10.86	2.168	0.096
DBP (mmHg)	75.25 ± 12.26	66.55 ± 10.13^*^	69.88 ± 7.25^*#^	66.50 ± 6.99^*&^	5.557	0.001
BMI (kg/m^2^)	24.42 ± 4.40	22.93 ± 5.89	22.39 ± 2.76	23.01 ± 2.98	1.738	0.163
Waist(cm)	84.00 ± 12.99	82.25 ± 11.89	79.52 ± 8.32	76.64 ± 7.42	2.173	0.095
WHR	0.87 ± 0.07	0.85 ± 0.07	0.84 ± 0.06	0.81 ± 0.04	2.861	0.054
BFR (%)	34.12 ± 6.21	30.15 ± 7.79^*^	32.41 ± 5.37^#^	30.03 ± 6.01^*&^	2.697	0.049
IL-6(pg/ml)	3.84 ± 2.27	2.58 ± 1.58^*^	3.67 ± 1.72^#^	2.52 ± 1.83^*&^	3.728	0.013
Hs-CPR (mg/L)	0.63 (0.40,1.60)	0.10 (0.10,2.22)	0.49 (0.29,0.94)	0.10 (0.10,0.40)	2.222	0.139
TNF-a(pg/ml)	10.51 ± 5.45	9.01 ± 5.39	10.17 ± 5.56	8.79 ± 4.78	0.556	0.645
TNF-β(pg/ml)	19.63 ± 6.60	13.64 ± 9.53	18.46 ± 7.21	13.21 ± 8.30	1.687	0.174
TC (mmol/L)	5.16 ± 0.86	4.79 ± 0.76^*^	4.61 ± 0.62^*^	4.48 ± 0.84^*^	3.680	0.014
TG (mmol/L)	1.67 ± 0.60	1.23 ± 0.93	1.00 ± 0.54	1.12 ± 0.61	2.045	0.111
HDL-C(mmol/L)	1.37 ± 0.30	1.29 ± 0.38	1.36 ± 0.30	1.37 ± 0.28	0.160	0.923
LDL-C(mmol/L)	3.12 ± 0.82	2.87 ± 0.47^*^	2.73 ± 0.54^*^	2.70 ± 0.72^*^	2.557	0.049
Lp(a) (mg/dl)	171.62 ± 99.81	124.45 ± 79.16^*^	112.86 ± 72.43^*^	111.55 ± 69.76^*^	1.639	0.018
FBG (mmol/L)	6.39 ± 2.21	6.18 ± 2.60	5.26 ± 0.45^*#^	5.01 ± 0.47^*#^	7.277	<0.001
HbA1c (%)	5.95 ± 1.12	5.53 ± 0.25	5.51 ± 0.30	5.42 ± 0.28^*^	3.999	0.001
Cor(ug/dl)	10.50 ± 2.95	9.71 ± 3.42	8.21 ± 2.59^*#^	7.85 ± 3.80^*#^	3.487	0.018
HOMA-β	122.31 ± 63.52	126.63 ± 76.18	145.03 ± 76.13	150.09 ± 109.87	0.734	0.534
HOMA-IR	4.84 ± 2.85	3.50 ± 2.76	2.38 ± 1.66^*#^	2.51 ± 1.50^*#^	4.765	0.004
TyG Index	2.08 ± 1.10	1.87 ± 1.22	1.41 ± 0.93^*#^	1.43 ± 0.91^*#^	3.464	0.019
Matsuda Index	6.95 ± 4.35	9.59 ± 5.47	13.75 ± 7.46^*#^	15.01 ± 7.46^*#^	11.011	<0.001

^*^p<0. 05*vs.*GDM-GMA Group; ^#^p<0. 05*vs.*Non-GDM-GMA Group; ^&^p<0. 05*vs.*GDM-NGT Group.

GDM, gestational diabetes mellitus; GMA, glucose metabolism anomaly; NGT, normal glycemic tolerance; SBP, systolic blood pressure; DBP, diastolic blood pressure; BMI, body mass index; WHR, waist-hip ratio; BFR, body fat rate; Cor, cortisol; FBG, fasting blood glucose; HbA1c, hemoglobin; TC, total cholesterol; TG, triglycerides; HDL-C, high-density lipoprotein cholesterol; LDL-L, low-density lipoprotein cholesterol; Lp(a), Lipoprotein (a); Hs-CRP, high sensitivity C-reactive protein; IL-6,interleukin-6; TNF-a, tumor necrosis factor-a; TNF-β, tumor necrosis factor-β; HOMA-β, homeostasis model assessment for β-cell function; HOMA-IR, homeostasis model assessment for insulin resistance; TyG, triglyceride glucose.

### 3.2 CGM in pregnant women 4–7 years postpartum

**Table 2** summarizes the CGM results for women 4–7 years postpartum. Regardless of GDM status, the GMA group had a higher mean blood glucose (MBG) level than the NGT groups [(6.31 ± 1.97,5.75 ± 0.59)vs. (5.35 ± 0.89,5.41 ± 0.51),p=0.004]. The GDM-GMA subgroup exhibited the highest maximum blood glucose (Max BG) levels among all groups[(11.28 ± 3.24)vs. (8.96 ± 1.56,9.66 ± 2.84,8.23 ± 1.23),p<0.001], even within the NGT group, women with prior GDM displayed elevated Max BG levels compared to their non-GDM counterparts(9.66 ± 2.84vs.8.23 ± 1.23,p< 0.001].

**Table 2:** Continuous blood glucose monitoring in pregnant women 4–7 years postpartum.

Metric Category	Characteristic	GDM-GMA	Non-GDM-GMA	GDM-NGT	Non-GDM-NGT	*F*	*P*
Glucose Levels	TIR (%)	94.49 ± 17.67	95.03 ± 11.01	97.78 ± 5.96	98.32 ± 0.94	1.269	0.288
	MBG (mmol/L)	6.31 ± 1.97	5.75 ± 0.59	5.35 ± 0.89^*#^	5.41 ± 0.51^*#^	4.738	0.004
	Max BG (mmol/L)	11.28 ± 3.24	8.96 ± 1.56^*^	9.66 ± 2.84^*^	8.23 ± 1.23^*&^	11.986	<0.001
	Min BG (mmol/L)	3.63 ± 1.64	3.84 ± 0.72	3.59 ± 0.82	3.66 ± 0.83	1.594	0.195
Glycemic Variability	SD (mmol/L)	1.22 ± 0.52	0.76 ± 0.27^*^	1.01 ± 0.49^*#^	0.71 ± 0.22^*&^	12.931	<0.001
	CV (%)	19.71 ± 6.86	13.14 ± 3.79^*^	18.93 ± 6.98^#^	13.19 ± 4.31^*&^	11.788	<0.001
	MAGE (mmol/L)	2.88 ± 1.36	1.89 ± 0.87^*^	2.15 ± 0.75^*^	1.61 ± 0.53^*&^	13.607	<0.001
	LAGE (mmol/L)	7.54 ± 2.77	4.72 ± 1.70^*^	6.56 ± 3.08^#^	4.56 ± 1.71^*&^	12.243	<0.001
	MODD (mmol/L)	1.08 ± 0.41	0.66 ± 0.26^*^	0.87 ± 0.28^*^	0.64 ± 0.19^*&^	16.664	<0.001
	ADRR (mmol/L)	0.92 ± 0.29	0.39 ± 0.24^*^	0.76 ± 0.28^#^	0.39 ± 0.13^*&^	26.232	<0.001

^*^p<0. 05*vs.*GDM-GMA Group; ^#^p<0. 05*vs.*Non-GDM-GMA Group; ^&^p<0. 05*vs.*GDM-NGT Group.

MBG, mean blood glucose; TIR, time in target range; SD, standard deviation; CV, coefficient of variation; MAGE, mean amplitude of glycemic excursion; LAGE, largest amplitude of glycemic excursions; Max BG, maximal blood glucose; Min BG, minimum blood glucose; MODD, mean of daily differences; ADRR, average daily risk range.

GMA subgroups with GDM history displayed significantly increased variability indices: Standard deviation (SD)(1.22 ± 0.52vs.0.76 ± 0.27,p<0.001), Coefficient of variation (CV) (19.71 ± 6.86vs.13.14 ± 3.79,p<0.001), Mean amplitude of glycemic excursions (MAGE) (2.88 ± 1.36vs.1.89 ± 0.87,p<0.001), Largest amplitude of glycemic excursions (LAGE) (7.54 ± 2.77vs.4.72 ± 1.70,p<0.001), Mean of daily differences (MODD) (1.08 ± 0.41vs.0.66 ± 0.26,p<0.001)and Average daily risk range (ADRR) (0.92 ± 0.29vs.0.39 ± 0.24,p<0.001). Even women with NGT but a history of GDM showed greater GV [SD(1.01 ± 0.49vs.0.71 ± 0.22,p<0.001), CV(18.93 ± 6.98vs.13.19 ± 4.31,p<0.001), MAGE(2.15 ± 0.75vs.1.61 ± 0.53,p<0.001), LAGE(6.56 ± 3.08vs.4.56 ± 1.71,p<0.001), MODD(0.87 ± 0.28vs.0.64 ± 0.19,p<0.001), ADRR(0.76 ± 0.28vs.0.39 ± 0.13,p<0.001)] over the 4–7 years postpartum. The GDM-GMA subgroup demonstrated the most pronounced SD, followed by GDM-NGT, then the non-GDM group[(1.22 ± 0.52)vs. (1.01 ± 0.49)vs. (0.76 ± 0.27,0.71 ± 0.22),p<0.001]. Women with prior GDM (regardless of current GMA status) exhibited higher LAGE [(7.54 ± 2.77,6.56 ± 3.08)vs. (4.72 ± 1.70,4.56 ± 1.71),p<0.001]and ADRR[(0.92 ± 0.29,0.76 ± 0.28)vs.(0.39 ± 0.24,0.39 ± 0.13),p<0.001]compared to non-GDM groups. There was no statistically significant difference in GV between the non-GDM subgroups, regardless of whether they developed GMA (p< 0.001). After adjusting for postpartum BMI, the intergroup differences in glycemic variability parameters remained significant (all p< 0.05).

### 3.3 Baseline characteristics of pregnant women in the perinatal period

**Table 3** compares the baseline characteristics of women in the GMA and NGT groups during the perinatal period. The pre-pregnancy BMI of the GMA group was significantly higher than that of the NGT group (25.52 ± 3.23 vs. 22.12 ± 3.12, p< 0.001). The uric acid (UA) level in the GMA group was higher than in the NGT group (233.59 ± 53.94 vs. 211.17 ± 58.47, p = 0.040). The incidence of GDM in the GMA group was significantly higher than that in the NGT group (79.5% vs. 32.9%, p< 0.001), and FBG was also elevated (5.21 ± 0.62 vs. 4.86 ± 0.40, p< 0.001). Similarly, the IR marker TyG index was significantly higher in the GMA group (1.27 ± 0.30 vs. 0.83 ± 0.49, p< 0.001) compared to the NGT group.

**Table 3:** Baseline characteristics of pregnant women in the perinatal period.

Characteristic	GMA-Group		NGT-Group		*F/H/χ2*	*P*
Age(years)	31.40 ± 3.84		31.56 ± 3.49		1.580	0.820
SBP (mmHg)	112.18 ± 11.36		108.57 ± 11.88		0.408	0.107
DBP (mmHg)	68.68 ± 10.77		67.45 ± 14.05		0.518	0.616
BMI-pre (kg/m^2^)	25.52 ± 3.23		22.12 ± 3.12		0.509	<0.001
GWG (kg)	11.80 ± 4.75		12.62 ± 4.20		0.124	0.327
Parity≥1	21	47.7%	33	43.4%	0.209	0.648
Gestational weeks	38.43 ± 1.48		38.28 ± 2.01		0.253	0.683
GDM	35	79.5%	25	32.9%	24.258	<0.001
WBC (10^9^/L)	8.34 ± 3.47		7.24 ± 2.26		8.551	0.138
PLT (10^9^/L)	250.05 ± 79.66		240.86 ± 45.03		2.229	0.421
HB(g/L)	130.13 ± 9.69		128.82 ± 11.46		0.880	0.526
NEU (10^9^/L)	6.24 ± 2.17		8.39 ± 4.16		1.210	0.576
LYM (10^9^/L)	1.85 ± 0.52		1.89 ± 0.55		0.045	0.733
MON (10^9^/L)	0.41 ± 0.17		0.41 ± 0.15		1.885	0.893
Hs-CRP (mg/L)	0.37 (0.10,0.79)		0.27 (0.10,2.11)		-0.176	0.860
Ferritin(ng/ml)	61.80 (50.95,61.80)		61.80 (47.40,61.8)		-1.084	0.278
ALB(g/L)	43.27 ± 3.01		43.34 ± 2.42		1.256	0.887
ALT(U/L)	13.02 ± 5.47		12.68 ± 4.45		0.659	0.847
AST(U/L)	20.07 ± 10.01		19.19 ± 10.61		0.024	0.793
sCr(umol/L)	50.68 ± 9.04		50.40 ± 9.01		0.004	0.870
UA (umol/L)	233.59 ± 53.94		211.17 ± 58.47		0.051	0.040
Hcy(umol/L)	6.14 ± 1.89		6.16 ± 1.75		0.380	0.970
TSH (uIU/ml)	1.71 ± 1.01		1.69 ± 1.15		0.273	0.932
FT3(pmol/L)	4.69 ± 0.61		6.60 ± 1.54		2.184	0.413
FT4(pmol/L)	17.26 ± 2.08		17.63 ± 5.04		2.041	0.644
TPOAb(positive)	7	15.9%	10	13.2%	0.173	0.677
FBG (mmol/L)	5.21 ± 0.62		4.86 ± 0.40		3.857	<0.001
TC (mmol/L)	4.12 ± 0.66		3.85 ± 0.95		2.275	0.109
TG (mmol/L)	1.15 ± 0.92		1.07 ± 0.45		3.172	0.604
HDL-C(mmol/L)	1.39 (1.21,1.55)		1.39 (1.20,1.82)		-0.202	0.840
LDL-C(mmol/L)	2.20 (1.88,2.60)		2.09 (1.66,2.51)		-1.372	0.170
TyG Index	1.27 ± 0.30		0.83 ± 0.49		0.284	<0.001

GMA, glucose metabolism anomaly; NGT, normal glycemic tolerance; SBP, systolic blood pressure; DBP, diastolic blood pressure; BMI-pre, Pre-pregnancy body mass index; GWG, gestational weight gain; GDM, gestational diabetes mellitus; WBC, white blood cell count; PLT, platelet; HB, hemoglobin; NEU, neutrophil; LYM, lymphocyte; MON, monocyte; Hs-CRP, high sensitivity C-reactive protein; ALB, albumin;ALT, alanine aminotransferase; AST, aspartate transaminase; sCr, serum creatinine; UA, uric acid; Hcy, homocysteine; TSH, thyroid stimulating hormone; FT3, free triiodothyronine; FT4, free tetraiodothyronine; TPOAb, thyroid peroxidase antibody; FBG, fasting blood glucose; TC, total cholesterol; TG, triglycerides; HDL-C, high-density lipoprotein cholesterol; LDL-L, low-density lipoprotein cholesterol; TyG, triglyceride glucose.

### 3.4 Analysis of maternal risk factors for the development of GMA in pregnant women 4–7 years postpartum

**Figure 3:**
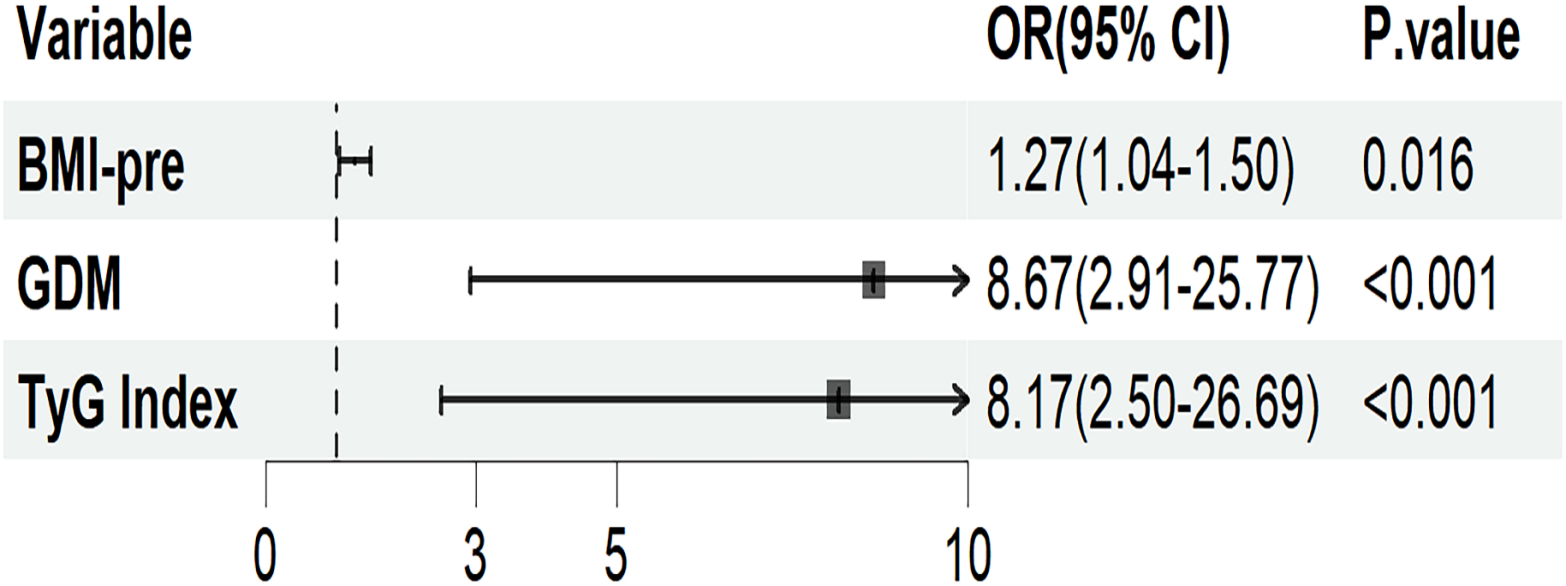
Logistic regression analysis of maternal risk factors for the onset of GMA in pregnant women 4–7 years postpartum. Adjusted for age, weight-add, parity, and gestational weeks. BMI-pre, Pre-pregnancy body mass index; GDM, gestational diabetes mellitus; TyG, triglyceride glucose; OR, odds ratio; CI, confidence interval.

**Figure 3** presents a logistic regression model with GMA as the dependent variable and the statistically significant indicators from univariate analysis as independent variables. After adjusting for age, weight-add, parity and gestational weeks, the results showed that pre-pregnancy BMI (OR = 1.27; 95% CI: 1.04-1.50), a history of GDM (OR = 8.67; 95% CI: 2.91-25.77), and TyG index (OR = 8.17; 95% CI: 2.50-26.69) are independent risk factors for the development of GMA in women 4–7 years postpartum (p< 0.05).

**Figure 4:**
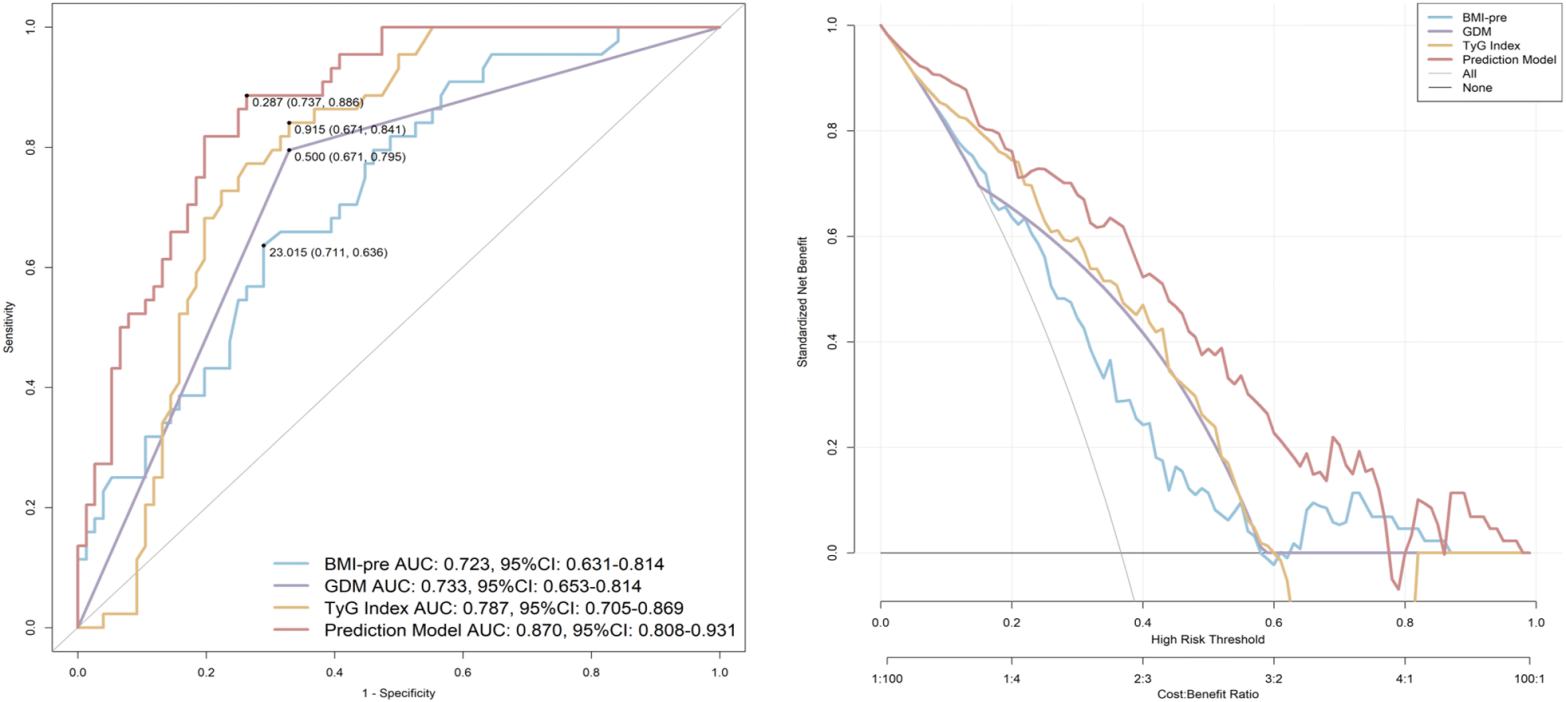
ROC curves to assess the predictive value of indicators for GMA 4–7 years postpartum. BMI-pre, Pre-pregnancy body mass index; GDM, gestational diabetes mellitus; TyG, triglyceride glucose; AUC, area under the curve; CI, confidence interval.

**Figure 4** evaluates the predictive performance of each indicator using ROC curves. The AUC for pre-pregnancy BMI was 0.723 (95% CI, 0.631-0.814), with a sensitivity of 71.1% and specificity of 63.6% at the optimal cutoff value of 23.015. The AUC for GDM was 0.733 (95% CI, 0.653-0.814), with a sensitivity of 67.1% and specificity of 79.5%. The AUC for the TyG index was 0.787 (95% CI, 0.705-0.869), with a sensitivity of 67.1% and specificity of 84.1% at the optimal cutoff value of 0.915. A predictive model named “Prediction” was established based on the three aforementioned risk factors. The AUC of this model was 0.870 (95% CI, 0.808-0.931), with a sensitivity of 73.7% and specificity of 88.6%. Decision curve analysis (DCA) further confirmed that this model offers the optimal clinical net benefit.

## 4 Discussion

This study found that women with a history of GDM had a significantly increased risk of developing GMA within 4–7 years postpartum (58.3% vs. 15.0%, p< 0.001). Among them, 18.3% developed T2DM, 35.0% had IGT, and 5.0% had IFG. These findings are consistent with the HAPO follow-up study, which also indicated that women with GDM remain at a higher risk of developing T2DM years after pregnancy (5). Furthermore, this study confirmed that women with a history of GDM had a 5- to 6-fold increased risk of developing postpartum T2DM, a finding consistent with the meta-analysis by Vounzoulaki et al (6). Several studies (13–15) have reported that the prevalence of GMA in women with GDM can range from 29% to 67% in the early (4–12 weeks) to mid-term (approximately 33 months) postpartum follow-up. In this study, the prevalence of GMA was 58.3% in women with GDM in the Chinese population up to 4–7 years postpartum, which is consistent with the trend of previous studies, and further revealed the cumulative effect of the risk at more distant follow-up. The results suggest that even with normal results on early postnatal glucose screening, women with GDM remain at significantly elevated metabolic risk over time, and the prevalence of GMA continues to increase over time. This finding further highlights the need to expand the metabolic management of the GDM population from short-term postnatal review to a systematic long-term follow-up mechanism for early warning and effective intervention of T2DM.

The GDM-GMA group exhibited a higher cardiometabolic risk, characterized by elevated DBP, BFR, IL-6, TC, LDL-C, and Lp(a) levels. Even in the NGT state, women with a history of GDM still exhibited higher cardiometabolic risk, primarily reflected in elevated DBP, BFR, and IL-6 levels. Studies have found that women with GDM maintain a heightened inflammatory state years after delivery, regardless of whether they develop postpartum GMA (16). The findings of this study, particularly the elevated IL-6 levels, further support this perspective. Participants in the GMA group exhibited higher FBG levels, accompanied by elevated cortisol levels. Additionally, women who developed GMA 4–7 years postpartum primarily exhibited greater IR, as indicated by higher HOMA-IR, TyG index, and Matsuda index, while β-cell function showed no significant difference between groups. Research suggests that the progression from GDM to T2DM and cardiovascular disease (CVD) in postpartum women is a dynamic process driven by shared pathogenic mechanisms, with chronic inflammation often being an early feature (17–20). The development of GDM may originate from an abnormal maternal immune adaptation to pregnancy and an upregulation of circulating inflammatory factors (21, 22), leading to immune pathway dysregulation. This, in turn, activates multiple metabolic pathways, promoting hyperinsulinemia and peripheral IR, accompanied by endothelial dysfunction and vascular lesions. Ultimately, this process progresses from glucose intolerance, hypertension, and dyslipidemia to atherosclerosis, and eventually to T2DM and CVD (7, 23–26). The findings of this study further confirm previous research while also identifying elevated Lp(a) levels, which may provide new insights into the atherosclerotic risk associated with GDM. In conclusion, the results of this study suggest that women with GDM may face an increased risk of CVD, further emphasizing the necessity of early intervention. It is recommended to enhance postpartum cardiovascular risk assessment and management to ensure continuous monitoring.

This study further revealed through CGM that GV was significantly elevated in the GDM subgroup. Even women in the GDM-NGT group exhibited greater GV (e.g., SD, CV, MAGE, LAGE, MODD, ADRR), suggesting that traditional HbA1c and OGTT may underestimate the early stages of metabolic dysregulation. This study found no statistically significant differences in TIR between groups, with glucose abnormalities primarily manifesting as increased GV. This may be because, in the 4–7 years postpartum period, IR is the predominant feature in women with GDM, while potential β-cell dysfunction has not yet become clinically evident. This characteristic aligns with the progression of T2DM (27). Studies have confirmed that GV is a core indicator of diabetes management, independent of HbA1c, and is closely associated with acute and chronic complications, cardiovascular risk, and patient quality of life (28–30). The potential mechanisms include GV accelerating β-cell apoptosis, exacerbating insulin secretion defects, and further promoting IR. Additionally, by increasing oxidative stress and inflammatory responses, GV may cause more severe endothelial cell damage than persistent hyperglycemia, accelerating atherosclerosis and leading to both microvascular and macrovascular complications. Moreover, it may also induce mitochondrial dysfunction, aggravating peripheral neuropathy. Previous studies have rarely focused on GV in postpartum women with GDM. This study provides new evidence through CGM, suggesting that CGM may serve as a more sensitive diagnostic tool than conventional OGTT for the early detection of metabolic abnormalities. The findings of this study support the perspectives of some researchers regarding the potential value of CGM in the early management of T2DM (8, 31). Furthermore, they suggest that CGM can serve as an early screening tool for identifying potential GMA, thereby reducing the long-term risk of T2DM and CVD.

This study found that pre-pregnancy BMI, the TyG index, and a history of GDM are independent predictors of GMA (AUC = 0.870). The predictive value of pre-pregnancy BMI (AUC = 0.723) aligns with global obesity trends (32, 33), further emphasizing the importance of pre-pregnancy weight management. The TyG index (AUC = 0.787), as a surrogate marker of IR (34), has particularly strong predictive value in Asian populations due to their heightened susceptibility to visceral fat accumulation. This finding also aligns with the previous research conducted by our group (9). This study developed a predictive model based on pre-pregnancy BMI, the TyG index, and a history of GDM, achieving an AUC of 0.870. The model demonstrated high predictive performance, providing a scientific basis for early intervention. For high-risk individuals with a pre-pregnancy BMI ≥23 kg/m², TyG ≥0.915, and a history of GDM, lifestyle interventions should be initiated as early as possible.

This study has certain limitations. Due to the single-center design and relatively small sample size, the generalizability of the study findings may be limited. Therefore, future multi-center, large-scale cohort studies are needed to further validate the stability and generalizability of these findings. The exclusion of potential influencing factors such as postpartum weight changes and breastfeeding duration may weaken the reliability of causal inferences to some extent. The current follow-up period of 4–7 years is still considered mid-to-short term. Thus, extending the follow-up period to over 10 years is necessary to comprehensively observe the natural progression of T2DM.

## 5 Conclusion

Women with a history of GDM exhibit greater GV within 4–7 years postpartum, accompanied by more pronounced cardiovascular risk factors. Pre-pregnancy BMI, TyG index, and a history of GDM are key independent predictors of GMA within 4–7 years postpartum. These findings underscore the critical role of continuous monitoring and early intervention in reducing the risk of long-term metabolic abnormalities and CVD.

## Data Availability

The raw data supporting the conclusions of this article will be made available by the authors, without undue reservation.
